# New ionizable lipids reduce the lipid-to-mRNA ratio for base editing

**DOI:** 10.1093/nsr/nwae224

**Published:** 2024-07-09

**Authors:** Shuliang Gao, Qiaobing Xu

**Affiliations:** Department of Biomedical Engineering, Tufts University, USA; Department of Biomedical Engineering, Tufts University, USA

CRISPR-Cas9-based genome editors are widely explored for the treatment of various diseases [1]. As part of CRISPR technology, base editors have been expanded to A-to-B (where ‘B’ = G, C or T), T-to-S (where ‘S’ = C or G), C-to-K (where ‘K’ = G or T) and G-to-Y (where ‘Y’ = C or T) conversions for generating precise site-specific point mutations without double-strand breaks (DSBs) [2]. Both delivery and RNA technologies have played significant roles in developing CRISPR-based therapeutics [3–5]. As a synthetic material-based delivery system, lipid nanoparticles (LNPs) can carry large cargoes and be optimized for immune compatibility to offer high levels of flexibility and control [1,6,7]. As a key LNP component, ionizable lipids can potentially cause immune toxicity (e.g. induce higher amounts of pro-inflammatory cytokines and chemokines) [8]. These immune responses can be reduced or eliminated by optimizing the design of the ionizable lipid, or by decreasing their amount in LNP formulations [8,9].

**Figure 1.**
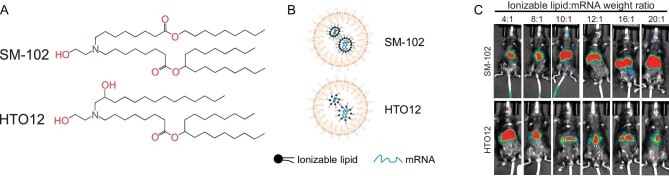
(A) Chemical structures of ionizable lipid HTO12. (B) Schematic illustration of LNP formulations. (C) *In vivo* whole-body luminescence imaging after delivery of SM-102 and HTO12. Reprinted from Ref. [9].

Recently, a team led by Professor Hao Yin at the Medical Research Institute, Wuhan University published a work in *National Science Review* [9] (Fig. 1) reporting a new ionizable lipid HTO12 with a higher count of hydroxyl groups compared with the commercial lipid SM-102, which enabled *in vivo* base editing of the mouse *Pcsk9* gene by delivering adenine base editor (ABE) mRNA and sgRNA with 2.5-fold reduction of the lipid/mRNA ratio compared with the SM-102 formulation.

In this study, three new candidate ionizable lipids (HTO12, HTO14 and HTO16) with various side chain lengths were synthesized using epoxide ring-opening reactions. These lipids contain hydroxyl groups on the alkyl chain. Then, they evaluate the mRNA loading and encapsulation efficiencies of these lipids at lipid/mRNA ratios ranging from 2:1 to 20:1 in an LNP formulation with necessary excipients. Compared with SM-102, HTO lipids showed similar encapsulation and higher loading efficiency, which may be attributed to an increase in the hydrogen bonding that formed between the lipids with mRNA due to the additional hydroxyl group in the HTO lipids. *In vivo* screening of formulations with firefly luciferase mRNA was then performed via chemiluminescence imaging. The HTO12 lipid formulation showed liver-targeting abilities and similar performance (e.g. luciferase translation levels, storage stabilities and biosafety profile) to SM-102. Notably, the amount of ionizable lipid was reduced by 2.5-fold in HTO formulations compared with SM-102 formulations.

As an example of therapeutic applications, HTO12 was used to deliver ABE components *in vivo* targeting the *Pcsk9* gene to reduce plasma cholesterol levels. HTO12 delivery enabled similar *Pcsk9* editing efficiency and decreased cholesterol levels on a par with SM-102.

In summary, this study developed a novel ionizable lipid that contains multiple hydroxyl groups that allow efficient delivery of various types of mRNA *in vivo* while substantially reducing the amount of ionizable lipid in the LNP formulation. The understanding obtained in this study will assist in the development of safer carriers for CRISPR-based therapeutics.
